# Skeletal muscle: a biologists’ adventure playground

**DOI:** 10.1007/s10974-025-09697-9

**Published:** 2025-06-02

**Authors:** Terence Partridge

**Affiliations:** Dubowitz Neuromuscular Centre, UCL Great Ormond Street Institute of Child Health, University College London, 30 Guilford Street, WC1N 1EH London, UK

**Keywords:** Skeletal muscle, Dystrophin, Satellite cells, Muscular dystrophy

## Abstract

A brief discussion about skeletal muscle, aberrant expression of dystrophin from null mutations of the gene, potential explanations as to why this occurs, and how understanding this could be useful for potential therapies in the future.

Scientists, like the generality of children, are compulsively drawn to the lure of mystery and conundrum, especially when associated with access to a variety of shiny toys. Just such a combination has fuelled popularity of skeletal muscle as a research topic, further fostered by the availability of an ever-increasing armoury of tools and reagents across the array of biological disciplines. Early interest in this tissue derives from its mechanical and, at a more mundane commercial level, its culinary, qualities but detailed scientific inspection has revealed an intriguing succession of strata of intimate relationships linking function to structure. These provide a rich playground for investigators across scales ranging from the mechanics and energetics of actin and myosin interaction during muscle contraction, through the cell biological basis of development, growth and maintenance, to the central place of muscle energetics in whole body metabolism, and progressively into the socio-economics of body enhancement and athletic performance.

The apparatus of contraction operates at high velocities and, on the cellular scale, over long distances. This entails the formation and strict spatially accurate organization of large linearly structured proteins arranged to act in concert to produce a highly oriented longitudinal contraction. Marriage of such a mechanically constraining architecture to the minimal diffusional obstruction required for efficient energy output from a robust metabolic mechanism, is reflected in the spectacular striped microscopic appearance that is a hallmark of skeletal muscle.

In the context of the seeming simplicity and structural regularity of skeletal muscle, the progressive development of technical advances has revealed a complexity of interaction between intercellular and extracellular structural components of muscle and increasingly too, of non-muscle tissues. Together, these demand radical revision of interpretation with which we are only gradually coming to terms. At the same time, it is becoming clear that, at the local level of gene expression and protein interaction, our mechanistic models lie at the margins of experimental verifiability. Skeletal muscle contains some of the largest known genes that encode some of the largest known proteins, whose precise positioning is fundamental to the structure and mechanical function of the tissue. The mechanisms by which such a high level of organization is maintained during repair, or adaptation to change of functional demand within such a mechanically hyperactive a tissue, is a challenge to the imagination.

Medical interest arises mainly from the essential role of skeletal muscle as the effector of individual mobility of large animals, both vertebrate and invertebrate. This fosters a strong interest in the fundamental mechanisms of muscle function and, in the context of human medicine, of failure of function in disease and during aging. In this endeavour the structural basis of function and dysfunction as revealed by histopathological analysis has, historically, played a predominant primary role (Dubowitz and Oldfors 2013). Such investigations have, been transformed by availability of an array of technological tools emerging from revolutionary advances in molecular biology: notably an ever-increasing stable of monoclonal antibodies and antisense agents to identify the various types of cells and to destroy them or to disable specific functions. These have expanded the arena of playthings and provided unprecedented opportunities to design and perform incisive investigations into the molecular and cell biological bases of development and maintenance of skeletal muscle. Among the most valuable advances has been the identification of animal models carrying the genetic defects responsible for a variety of human myopathies. These have provided bases for investigation of the pathological processes behind each disease as well as material for preclinical testing of hypothetical therapeutic interventions. In our laboratory, Jenny Morgan inaugurated one of the dominant medical research themes in this tissue, namely investigation of the cellular mechanisms behind the processes of maintenance and repair of skeletal muscle, with emphasis on the failings of these mechanisms as a feature of myopathic conditions. Her early ground-breaking work on regeneration of skeletal muscle, including the role of a subset of adult muscle stem cells (Cousins et al. 2004; Beauchamp et al. 2000; Blaveri et al. 1999; Boldrin and Morgan 2013; Crist et al. 2009; Morgan and Partridge 2020; Morgan et al. 1990; Zammit et al. 2002), fuelled the idea of grafting myogenic precursor cells to supplement failing endogenous myogenic reserves whilst acting as vectors of corrected copies of mutant genes, such as that responsible for Duchenne muscular dystrophy (Beauchamp et al. 2000; Blaveri et al. 1999; Boldrin and Morgan 2012, 2013; Zammit et al. 2002; Boldrin et al. 2015; Collins et al. 2009; Partridge et al. 1989). Availability of anti-dystrophin antibodies was important for confirmation of success of these grafts but also identified unexpected features of the mechanisms responsible for splicing together the 79 exons of which this gene is composed. For, embarrassingly, dystrophic muscles, not grafted with normal myogenic cells, or where the grafted cells were themselves derived from dystrophic donors, contained occasional groups of dystrophin^+ ve^ muscle fibres. These muscle cells appeared to be disobeying the rules of molecular biology!

The finding of aberrant expression of the dystrophin protein from nominally null mutations of the gene, sparked a brief phase of investigation around the ‘revertant dystrophin’ phenomenon. Contemporaneous descriptions of a protein located at the surface of muscle fibres that stained strongly with anti-dystrophin antibodies were reported in muscle biopsies from Duchenne patients (Nicholson et al. 1993; Wallgren-Pettersson et al. 1993; Fanin et al. 1995), and in muscle samples from the mdx dystrophic mouse (Hoffman et al. 1990). In both human DMD and mdx mouse muscle biopsies, these dystrophin^+ ve^ fibres were disposed as scattered isolated groups. Immunostains with exon-specific monoclonal antibodies showed a pattern of epitope staining that varied between clusters within the same biopsy but was identical between all individual fibres within each cluster, and along each discrete immunostained segment within each fibre, indicating a clonal descent of each variant (Lu et al. 2000a, b). In the course of her satellite cell grafting experiments, Jenny Morgan had previously demonstrated similar localization of dystrophin within defined boundaries around myonuclei that had entered dystrophic fibres from grafts of genetically normal satellite cells (Blaveri et al. 1999).

The simplest explanation of the presence of a variety of clonal clusters that had escaped the mdx null mutation was that a second mutation had fortuitously restored open reading frame (Wallgren-Pettersson et al. 1993; Klein et al. 1992). In the mdx mouse models of DMD, the frequency of occurrence of these clusters varies between individual mdx mutant strains (Danko et al. 1992; Fritz et al. 1995), being highest in the originally described C5BL10 mdx strain where a point mutation introduces a stop codon into exon23, despite which, dystrophin positive ‘revertant’ muscle fibres are regularly seen in frozen sections. Such frequent occurrence of an unexpected phenomenon, in the context of a common genetic background of a well characterized and experimentally accessible model, invites deeper experimental interrogation. Expansion of the available range of monoclonal antibodies, permitted us to gather data that raises doubts about the ‘second mutation’ hypothesis. Mutant exon-23 in this mouse, lies upstream of 20 exons that retain open reading frame and several of these appear in different combinations within the range of epitope expression patterns that distinguish some 20 different types of clonal cluster in this animal (Lu et al. 2000a, b). Some of the variant patterns of epitope sequence comprising these quasi-dystrophins involve more than one discontinuity within the sequence of expressed exons, implying the formation of in-frame transcripts involving at least two splice-events within the predicted transcripts that would explain such epitope patterns. Double discontinuities of this sort have not been noted in the in-frame exon sequences seen in mutations associated with mutations associated with Becker’s Muscular dystrophy.

An alternative explanation would be some form of specific modulation of the transcription mechanism that promotes omission or inclusion of specific exons in a consistent manner within each individual muscle fibre, but in a different manner in different fibres. An adequate descriptive explanation would need to accommodate the fact that each clonal cluster, characterized by its individual variant pattern of exon expression within these quasi-dystrophins, be faithfully heritable over numerous serial divisions of the satellite cell in which the primary change had occurred. In new-born animals the revertant fibres were detected as rare, isolated single fibres or doublets of dystrophin^+ ve^ segments. With increasing age, each cluster showed an increase in number of constituent dystrophin^+ ve^ profiles together with the length of dystrophin positivity within each fibre. Importantly, individual epitope expression patterns were identical between all fibres within each cluster. Localization of revertant dystrophin is strongly suppressed by competition with co-expressed transgenic dystrophin (Crawford et al. 2001) or utrophin, as well as by doses of radiation sufficient to block regeneration, arguing that expansion of revertant dystrophin fibres with age is dependent on regeneration (Yokota et al. 2006; Rodrigues et al. 2016). Indeed, the persistent increase in cluster size indicates continuing regeneration from the same clonal source at around birth up to at least 18 months of age. This portrays the formation of revertant clusters as individual clonal events requiring that the satellite cells responsible for each clone continue to proliferate through much of the animal’s life. To transcribe a significant proportion of several different translatable transcripts, with different uniquely restored open reading frames, from a common genetic sequence would seem to require intervention of variables other than that present in the primary sequence. I am unaware of any proposed mechanisms that might cover these requirements; it might be better explained in terms of epigenetic factors that induce an array of different, discrete, highly reproducible, modifications of the splicing process around sites of mutations.

In addition to generating insights into the intimate corners of molecular biology, the clonal nature of expression of ‘revertant dystrophins’ also provides lineage markers that leave ‘snail trails’ marking migration paths of clones of myogenic cells and the revertant segments of muscle fibres to which they contribute. Changes in the size and distribution of these revertant fibre clusters over the lifespans of mdx mice provide unforeseen perspectives on the cellular biology of muscle regeneration. The protective effect of expression of a partially functional dystrophin molecule would be expected to selectively increase their proportion within the total population, if only subliminally, due to the cumulative absolute loss of dystrophin negative fibres. More interestingly, the gradual but continual absolute increase in the number of revertant fibres in each clonal cluster requires that the satellite cells carrying the revertant mechanism continue to proliferate and terminally differentiate throughout the period of growth and concomitant degeneration and regeneration (Lu et al. 2000a, b) in these dystrophic animals. This indicates a more-or-less lifelong continuation of expansion from clones established at birth together with persistence of replicative potential of these cells into old age. Maintenance of the compact structure of the progressively enlarging clusters suggests that the satellite cells responsible for the spread of ‘revertant’ dystrophin expression move actively between fibres, but almost exclusively between contiguous fibres. This is the most precise evidence of the nature of stem cell in vivo in muscle. Reports of the presence of large dystrophin-revertant clones in human DMD biopsies (Arechavala-Gomeza et al. 2010), suggest that similar mechanism are at work in man.

Thus far, this heritable mechanism of precise exclusion of specific sets of exons from individual transcripts has resisted all attempts at elucidation (Lu et al. 2000a, b) but the existence of such a mechanism does elicit some wider fundamental questions. In the specific case of dystrophin, function-restoring elimination of exons from the transcript is readily detectable, because it results in the restoration of expression of a protein that is functionally protective in a null background, thus providing a boost to identification of a clear signal. If this sort of perturbation were to occur in the context of a normal genomic sequence, it would tend to result in production of non-functional protein molecules and be undetectable, thus biasing to underestimates of the frequency of such impromptu events.

In the context of the mutated dystrophin gene, the restoration of production of a functional protein is too rare and functionally ineffectual an event to significantly mitigate the severity of the background pathology and thus exert selective evolutionary influence. It seems unlikely that the underlying mechanism is limited to its occasional aberrant role in rectifying in the production of dystrophin. It may be noted, however, that the mechanical role of skeletal muscle is dependent on many large linear proteins, such as actin, myosin and most spectacularly, titin, in which many allelic variants are generated by alternative splicing (Guo et al. 2010, 2012, 2018; Greaser and Pleitner 2014). Function of these proteins involves physical and biochemical interaction of dedicated domains with other proteins of the contractile apparatus (Dutta et al. 2023a, b; Tamborrini et al. 2023). These domains are linked by relatively uncommitted regions in which minor diversity is thought to contribute to diversity of function and in which factors other than the primary genetic sequence appear to play a role in the generation of this diversity (Guo et al. 2012). Current analytical approaches are designed to identify genetic sources of splice variants but are less likely to detect distinction between genetic and epigenetic mechanisms of the type that might explain ‘revertant’ expression of dystrophin (Morin et al. 2023).

Popularity of skeletal muscle among biochemists, molecular, and cell biologists is attributable to the treasure trove of highly organized proteins, and to the mechanisms that maintain this structure in the face of persistent mechanical and biochemical stress across a lifespan. As illustrated by the above example, even a chance encounter with an obscure deviation from ‘the expected’, can lead to a miscellany of unpredictable adventures into fundamental aspects of the biology of this tissue. Who could resist such a gift?

**Fig. 1:**
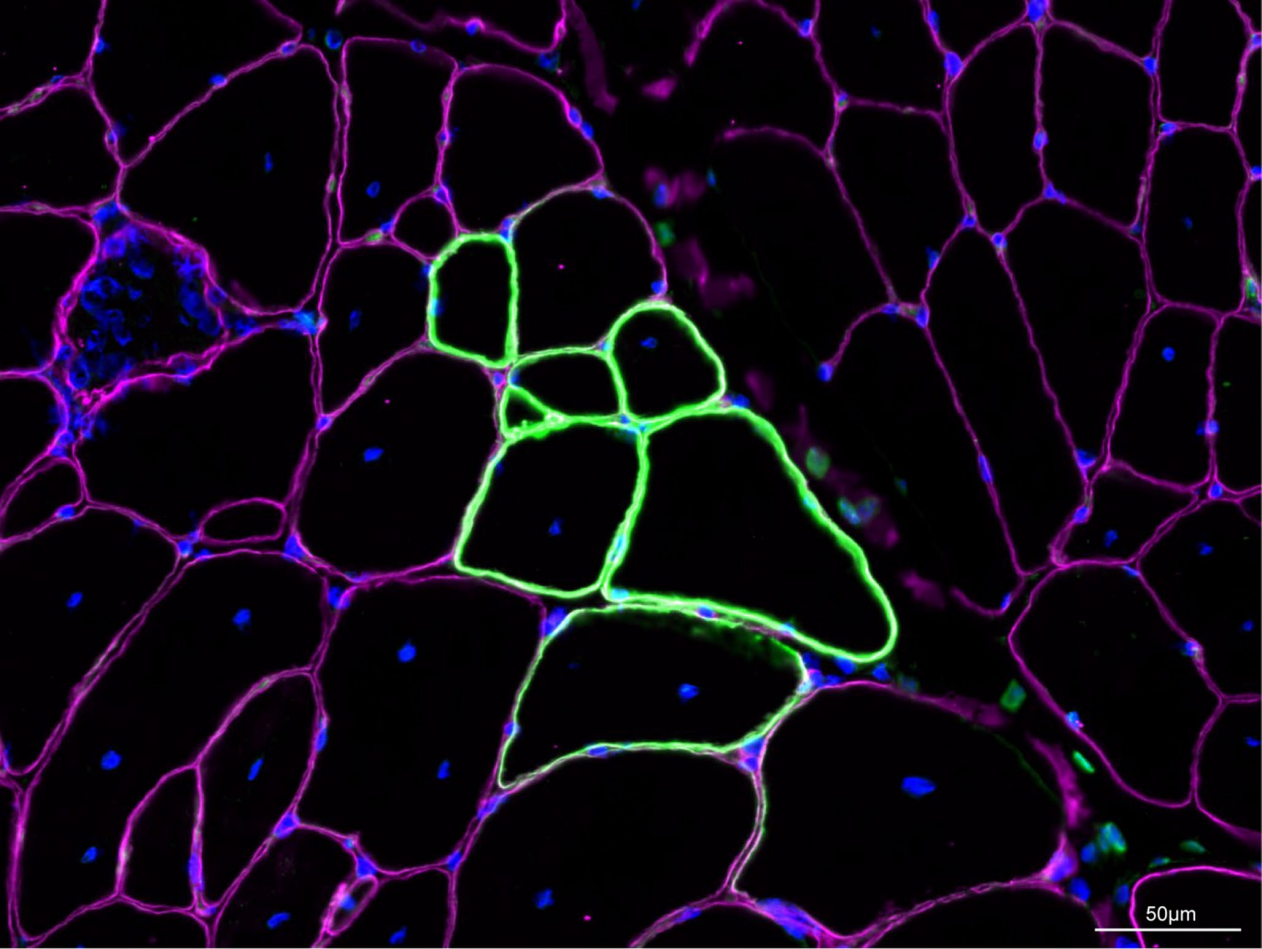
Revertant dystrophin-expressing fibers in B10-mdx muscle. Revertant dystrophin-expressing fibres (green) observed in B10-mdx (9.5 weeks old) tibialis anterior muscle. Muscle sections were stained with a rabbit anti-dystrophin polyclonal anti body followed by a fluorescein-conjugated mouse antirabbit monoclonal antibody and counterstained for laminin-α2 (pink) to delineate myofibers and with DAPI for nuclear detection. Scale bar represents 50 μm. Such fibres are detected in neonatal mdx mouse muscles as single fibres or doublets and increase with age up to tens or hundreds of fibres per cluster

## Data Availability

No datasets were generated or analysed during the current study.
