# Correction: Skeletal muscle: a biologists’ adventure playground

**DOI:** 10.1007/s10974-025-09714-x

**Published:** 2025-10-28

**Authors:** Terence Partridge

**Affiliations:** Dubowitz Neuromuscular Centre, UCL Great Ormond Street Institute of Child Health University College London, 30 Guilford Street, WC1N 1EH London, UK

**Correction to: Journal of Muscle Research and Cell Motility**

10.1007/s10974-025-09697-9

In this article, Author contributions statement was incorrectly given as TP wrote the manuscript. Michelle peckham is only submitting this manuscript on behalf of TP, as he is having difficulty doing so. Please do not consider MP as an author or as a contributor but it should have been removed.

The original article has been corrected.

